# Fibromyalgia: A Review of the Pathophysiological Mechanisms and Multidisciplinary Treatment Strategies

**DOI:** 10.3390/biomedicines12071543

**Published:** 2024-07-11

**Authors:** Lina Noelia Jurado-Priego, Cristina Cueto-Ureña, María Jesús Ramírez-Expósito, José Manuel Martínez-Martos

**Affiliations:** Experimental and Clinical Physiopathology Research Group CTS-1039, Department of Health Sciences, School of Experimental and Health Sciences, University of Jaén, E-23071 Jaén, Spainccueto@ujaen.es (C.C.-U.); mramirez@ujaen.es (M.J.R.-E.)

**Keywords:** fibromyalgia, quality of life, physiopathology, physical exercise

## Abstract

Fibromyalgia is a syndrome characterized by chronic widespread musculoskeletal pain, which may or may not be associated with muscle or joint stiffness, accompanied by other symptoms such as fatigue, sleep disturbances, anxiety, and depression. It is a highly prevalent condition globally, being considered the third most common musculoskeletal disorder, following lower back pain and osteoarthritis. It is more prevalent in women than in men, and although it can occur at any age, it is more common between the ages of thirty and thirty-five. Although the pathophysiology and etiopathogenesis remain largely unknown, three underlying processes in fibromyalgia have been investigated. These include central sensitization, associated with an increase in the release of both excitatory and inhibitory neurotransmitters; peripheral sensitization, involving alterations in peripheral nociceptor signaling; and inflammatory and immune mechanisms that develop concurrently with the aforementioned processes. Furthermore, it has been determined that genetic, endocrine, psychological, and sleep disorders may influence the development of this pathology. The accurate diagnosis of fibromyalgia remains challenging as it lacks specific diagnostic biomarkers, which are still under investigation. Nonetheless, diagnostic approaches to the condition have evolved based on the use of scales and questionnaires for pain identification. The complexity associated with this pathology makes it difficult to establish a single effective treatment. Therefore, treatment is multidisciplinary, involving both pharmacological and non-pharmacological interventions aimed at alleviating symptoms. The non-pharmacological treatments outlined in this review are primarily related to physiotherapy interventions. The effectiveness of physical exercise, both on land and in water, as well as the application of electrotherapy combined with transcranial therapy and manual therapy has been highlighted. All of these interventions aim to improve the quality of life of patients highly affected by fibromyalgia.

## 1. Introduction

Fibromyalgia is a chronic functional pathology characterized by widespread musculoskeletal pain, associated with heightened responses to stimuli perceived as nociceptive and linked to somatic symptoms [1,2]. Musculoskeletal pain is defined as persistent or recurrent pain in the bones, joints, muscles, or tendons lasting more than three months, categorizing it as chronic rather than acute pain [3,4,5]. In fibromyalgia, this pain may or may not be associated with joint stiffness [6], often accompanied by fatigue and sleep disturbances [7]. Chronic widespread musculoskeletal pain is classified into primary and secondary types [8]. Primary pain is experienced within the locomotor system and is characterized by functional limitation, whereas secondary pain is not only related to nociception but may also be associated with deep somatic injury [3]. This pathology exhibits some heterogeneity, as although its etiology is often unknown and no specific cause is identified in most patients, its onset may be associated with specific conditions such as infectious diseases [9], including parvovirus, brucellosis, or Lyme disease; diabetes [10]; rheumatic diseases [11]; and neurological disorders [1,12,13]. Regarding its symptoms, there is central sensitization triggering chronic pain, which may or may not be associated with joint and muscle stiffness [5,12]. Moreover, it presents somatic impairments including cognitive dysfunction, sleep and mood disorders, anxiety, depression, and fatigue, as well as general sensitivity, muscle tension, peripheral dysesthesia, and even inability to perform daily activities [7,8,12,14,15]. Currently, the exact cause of fibromyalgia is unknown, although various studies conducted in recent years in animals and humans have revealed metabolic [16], biochemical [17], genetic [18], and immunoregulatory abnormalities [1,19]. Despite being a fairly common syndrome in the population, there is no consensus regarding the diagnostic techniques to be implemented [20,21,22,23,24], nor for its classification and etiopathogenesis [22,25]. This complicates the application of effective treatment in affected individuals, reflected in the massive costs incurred in multidisciplinary medical care [25,26,27,28].

## 2. Epidemiology

**Figure 1:**
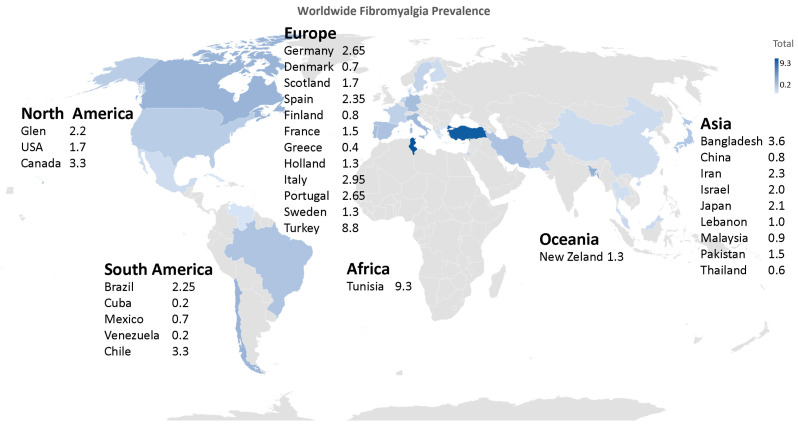
Worldwide prevalence of fibromyalgia.

Fibromyalgia is a highly prevalent syndrome in the general population, being considered the third most common musculoskeletal condition, following lower back pain and osteoarthritis [25,29,30]. The prevalence of fibromyalgia is a parameter that varies depending on the diagnostic criteria used for its detection. The most commonly used diagnostic criteria have been the 1990 American College of Rheumatology (ACR) criteria and the 2010 ACR criteria, as well as the use of various questionnaires for assessment and diagnosis of fibromyalgia conducted in different countries worldwide, resulting in different estimated prevalence values by different studies [25,29,30]. The estimated prevalence worldwide ranges from 2–3% [25,31] (Figure 1).

In a study conducted in five European countries—France, Portugal, Spain, Germany, and Italy—a prevalence of 4.7% and 2.9% was obtained in the overall European population based on two different sets of criteria [32]. Following this study, it was concluded that fibromyalgia appeared to be a common condition in these five countries [25,31,33]. In a multicenter population-based study, a prevalence of 2.45% was obtained in a Spanish adult sample, similar to the rate for the whole of Europe which was 2.64%. This study also observed that fibromyalgia is more common in women, with a mean age range of 60–69 years, and is primarily characterized by low socioeconomic status [31,34]. This syndrome is more common in women than in men, occurring in three women for every man. The age range in which it typically appears is between 30 and 35 years, although its peak prevalence is reached at between 50 and 60 years [25,35]. In Asian countries, FM has been reported to have varying prevalence rates. In South Korea, the prevalence of fibromyalgia was found to be 1.7% of the population, with a higher prevalence in women [36]. In China, a cross-sectional study reported a mean age of 49.4 years among patients with fibromyalgia, with a male-to-female ratio of 1:6.3 [37]. In Japan, a study found a mean age of 47.3 years among patients with fibromyalgia, with a male-to-female ratio of 1:8.1 [38].

## 3. Physiopathology

The pathophysiological factors of fibromyalgia remain subject to investigation as they are currently unknown. This pathology appears to be associated with a pain processing issue in the brain. In most cases, individuals affected by fibromyalgia tend to become hypersensitive to pain; this heightened pain vigilance may also be related to psychological issues [1,12].

The most significant alterations observed in fibromyalgia involve dysfunctions in monoaminergic neurotransmission, leading to elevated levels of excitatory neurotransmitters such as substance P [39,40] and glutamate [41], as well as decreased levels of serotonin and norepinephrine in the spinal cord at the level of descending antinociceptive pathways. Other anomalies such as dopamine dysregulation [42] and altered activity of endogenous brain opioids [43] have also been observed. Collectively, these cited phenomena appear to explain the central pathophysiology of fibromyalgia [12].

Over time, studies have identified pain generators as potential causes of fibromyalgia, and in these cases, patients often present symptoms related to cognitive impairment, sleep disturbances, chronic fatigue, mood disorders [5,6,7], and even intestinal irritability and interstitial cystitis [12,44].

### 3.1. Underlying Processes in Fibromyalgia

Several processes underlie fibromyalgia, a condition considered as a central sensitization syndrome. However, while central sensitization plays a significant role in this pathology, understanding the process of persistent nociceptive input related to tissue damage, as well as peripheral sensitization, is crucial [12,45]. Studies focused on peripheral pain generator blockade suggest that fibromyalgia symptoms should either disappear or not develop with such a blockade [12,46]. Nevertheless, researchers, lacking substantial evidence supporting the involvement of peripheral pain tissue anomalies and nociceptive processes in fibromyalgia, tend to emphasize the study of central sensitization [12,47]. However, sensitization is not a unitary process, thus necessitating a distinction between central, peripheral, and psychosocial sensitization. Central sensitization is defined as a mechanism amplifying neuronal signaling within the central nervous system (CNS), leading to increased pain perception. Individuals with fibromyalgia exhibit an expanded pain receptive field, hyperalgesia (increased pain perception to a non-painful stimulus), and allodynia (pain in response to a non-painful stimulus). This mechanism is also implicated in chronic and persistent pain [12,48]. Peripheral sensitization involves an augmented response from peripheral nociceptors, causing the neuronal system to increasingly respond to milder stimuli [48,49]. Psychosocial sensitization introduces two new terms: cognitive-emotional sensitization, explaining how selective attention to specific bodily pain can exacerbate the pain itself, and interpersonal sensitization, related to shared neuronal representation regarding the subject’s pain experience [12].

#### 3.1.1. Central Sensitization

Patients with fibromyalgia present a lower pain threshold, resulting in diffuse hyperalgesia and/or allodynia. This indicates potential issues with pain amplification or sensory processing in the CNS. These fibromyalgia phenomena have been confirmed in clinical studies utilizing functional neuroimaging or measuring alterations in neurotransmitter levels influencing sensory transmission and pain [12,14,47]. It has been observed that in fibromyalgia patients, the central sensitization process is characterized by increased neurotransmitter release. Among these neurotransmitters, substance P and glutamate activate NMDA receptors responsible for pain transmission. Substance P, released by specific sensory nerve terminals, binds to NK-1 receptors, with levels in fibromyalgia patients elevated up to three times above the normal level in cerebrospinal fluid [50]. This neuropeptide decreases the synaptic threshold of spinal neurons while being released through NMDA receptors in the dorsal horn of the spinal cord. Substance P travels along the spinal cord to sensitize distal neurons relative to the dorsal horn. It is also closely related to serotonin presence in the brain, particularly in areas associated with nociception and emotions [14,51]. On the other hand, several studies have shown that glutamate levels increase in fibromyalgia subjects following nociceptive stimulation [14,52].

##### Chronic Nociplastic Pain

The term “nociplastic pain” was introduced by the International Association for the Study of Pain (IASP) in 2017 as a third mechanistic descriptor of pain, alongside nociceptive and neuropathic pain. Nociplastic pain is defined as pain that arises from altered nociception, despite a lack of clear evidence of actual or threatened tissue damage causing the activation of peripheral nociceptors, or any disease or lesion of the somatosensory system. This term is intended for both clinical and research use to identify individuals experiencing pain and hypersensitivity in regions with apparently normal tissues and no signs of neuropathy. While central sensitization is likely a dominant mechanism in nociplastic pain conditions, the term should not be considered synonymous with the neurophysiological concept of “central sensitization”. Additionally, a contribution of peripheral sensitization cannot be ruled out. The concept of nociplastic pain aligns with the current understanding that certain forms of chronic pain are better viewed as conditions or diseases in their own right, rather than merely symptoms of other underlying pathologies. Chronic pain conditions such as fibromyalgia are a clear example where nociplastic pain is typically present. These conditions have documented changes in nociceptive processing within the nervous system, precluding the classification of their pain as “pain of unknown origin” (idiopathic pain). Clinical criteria define aspects that must be considered before assigning the descriptor nociplastic pain.

#### 3.1.2. Peripheral Sensitization

**Figure 2:**
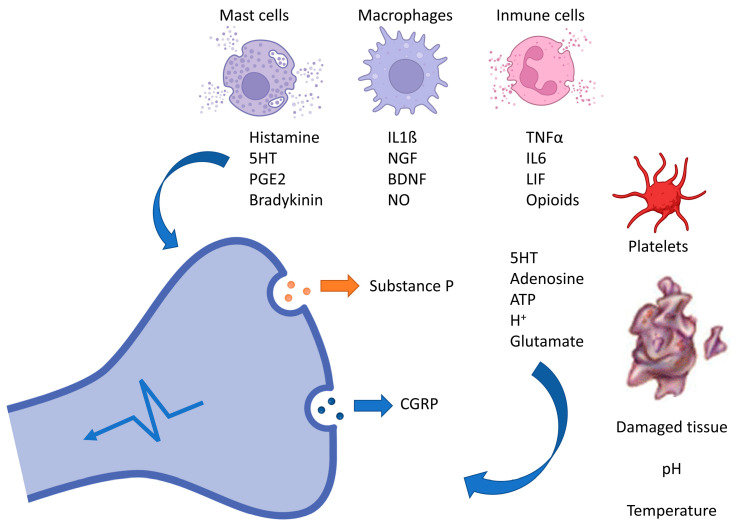
Mechanisms of peripheral sensitization in chronic pain. This diagram depicts the intricate interplay of biochemical and cellular components involved in peripheral sensitization during chronic pain. Key players include mast cells, macrophages, and immune cells, which release inflammatory mediators (such as histamine, IL-1β, and TNF-α). These substances interact with receptors on damaged tissue, influencing the release of neuropeptides such as substance P and CGRP. Ultimately, this altered response to pain contributes to the persistence of chronic pain states.

In the process of peripheral sensitization, nociceptors are nerve endings responsible for responding to mechanical, thermal, and chemical stimuli of a certain intensity, which connect with the dorsal horn of the spinal cord (CNS) via myelinated A-deltafibers and unmyelinated C fibers [45,48]. The arrival of nociceptive information at the dorsal horn of the spinal cord prompts neurons to release vesicles containing substance P, acting as a neuromodulator, along with other substances such as calcitonin gene-related peptide (CGRP) [46,48]. These substances are also released in the periphery, where they bind to receptors on cells associated with inflammatory processes such as mast cells, neutrophils, and basophils. This binding triggers the release of pro-inflammatory substances such as cytokines, chemokines, etc. [48,53], as illustrated in Figure 2 [48]. Stimulation of nociceptors leads to changes in the neuronal membrane’s electric charge, resulting in the propagation of nervous stimuli to the spinal cord, where glutamate, an excitatory amino acid, is released. Located in the postsynaptic neuron, glutamate conveys information to higher nuclei of the central nervous system (CNS) [45]. From these higher nuclei, descending pathways are activated, conveying the stimulus back to the dorsal horn of the spinal cord where the release of inhibitory substances takes place, acting on both glutamate release and hyperpolarization of the postsynaptic neuron membrane [16,45,48]. Therefore, in peripheral sensitization, two basic processes related to pain perception and its chronicity occur: an increase in the release of excitatory substances, primarily glutamate, and an enhancement in the transmission efficiency of nociceptive signals [48,53].

Several studies have demonstrated changes in peripheral nerves in patients with this pathology. These studies have been supported through skin biopsies, revealing a decrease in the number of epidermal fibers, and clinically in subjects presenting the pathology, obtaining higher scores in questionnaires related to neuropathic pain and the presence of alterations in perception thresholds for heat, cold, and pain [12]. Peripheral impulses are transmitted to the central nervous system through myelinated A-delta fibers and unmyelinated C fibers [12,45,48]. Myelinated A-delta fibers are activated by mechanical and thermal stimuli, transmitting pain signals rapidly and generating a short-lived painful sensation. These fibers, along with small unmyelinated C fibers, are predominantly located in superficial organs such as the skin [12,46]. C fibers are activated by thermal, mechanical, and chemical impulses, with pain signals traveling more slowly compared to myelinated A-delta fibers, resulting in a widespread painful sensation closely associated with fibromyalgia. These nerve fibers innervate deeper somatic organs such as joints and muscles, as well as the superficial organs mentioned earlier [12,45,46]. Additionally, they promote the release of cytokines, chemokines, and pro-inflammatory neuropeptides, thus being associated with peripheral neuroinflammation [12,45]. The physiological direction of nerve impulse transmission extends from peripheral areas, where the two aforementioned fiber types are located, to the spinal cord. However, there is also propagation of the signal in the opposite direction, leading the impulse to return to the junction points of the C fiber terminal, resulting in the subsequent release of pro-inflammatory substances. This process increases the neuron’s sensitivity to subsequent stimuli, amplifying it and giving rise to peripheral sensitization [12,14,45,46,53].

Additionally, there is increasing evidence of the potential role of peripheral mechanisms in the pathogenesis of fibromyalgia symptoms, with a subset of patients developing small fiber deficits. Combined with possible features of peripheral neuropathic pain including burning, paresthesia, hyperalgesia, and allodynia, the origin of pain may, in a certain proportion of people, stem from the peripheral nervous system. A systematic review and meta-analysis indicated small nerve fiber loss occurring in approximately 50% of patients with fibromyalgia [54]. As well structural alterations, abnormal spontaneous activity and/or sensitization of nociceptive C fibers have been identified in patients with fibromyalgia, suggestive of involvement in pain generation/maintenance. Additionally, it has been argued that abnormalities in pain evoked potentials, and quantitative sensory testing (QST) parameters suggest a potential peripheral mechanism, although this remains unclear [55].

#### 3.1.3. Inflammation

Despite the well-characterized relationship between the nervous system and inflammation, much of the mechanism linking different pathological features of fibromyalgia, including manifestations related to central sensitization, stress, and dysregulation of innate and adaptive immune responses, remains unknown [56,57]. The hypothalamic–pituitary–adrenal (HPA) axis plays a crucial role in the development of central sensitization [56,58]. It is responsible for controlling the stress response, and its activation leads to the secretion of corticotropin-releasing hormone (CRH), which modulates the immune response by releasing glucocorticoids. This includes increased mast cell infiltration expressing corticotropin-releasing hormone receptors; activated mast cells release granules that can induce sensitization at peripheral nociceptor and central levels and further enhance the release of pro-inflammatory cytokines associated with immune system activation [56,59]. Neurogenic inflammatory processes occurring in peripheral structures and within the central nervous system (spinal cord and brain) have been correlated through studies of fibromyalgia pathophysiology [58,60,61]. Moreover, the release of pro-inflammatory substances such as cytokines and chemokines leads to activation of the innate and adaptive immune system [12,57,58,60]. These processes manifest as numerous peripheral clinical characteristics present in patients with this condition, including inflammation and altered perception to tactile stimuli (dysesthesia), which may also affect central symptoms, including cognitive changes and fatigue. Relatively recent studies suggest that the development of fibromyalgia is preceded by hypothalamic inflammation, as elevated levels of IL-8 chemokines, biologically active pro-inflammatory substances, are found in serum and cerebrospinal fluid. IL-8 chemokines directly contribute to nociception by binding to chemokine receptors present along pain pathways [56]. Chemokines, along with cytokines, especially IL-1 beta, TNF alpha, IL-6, and IL-17, contribute to the central nervous system’s inflammatory response. Additionally, immune cells such as monocytes, neutrophils, and mast cells, as mediators of inflammatory processes, may play a role in defining an inflammatory substrate for fibromyalgia [56,58,60,61]. Pain associated with fibromyalgia is accompanied by neuroinflammatory processes triggered by microglia and mast cells [59]. Mast cells play a significant role in the development of conditions such as stress, depression, and anxiety in this pathology, as well as in pain conditions [59]. Activated microglia secrete chemokines and cytokines that contribute to a hyperactive state of neurons and central sensitization [56,58,60,61]. Hence, studies emphasize the role of immune cells and mediators in maintaining musculoskeletal pain and central sensitization [56,57,58,59,60,61].

## 4. Etiopathogenesis

Although the etiology of fibromyalgia remains unknown, various factors and alterations in neurotransmitters and genes have been studied, which collectively seem to explain the pathophysiology of fibromyalgia. Additionally, a study based on functional magnetic resonance imaging confirmed central neuronal alteration in nociceptive processes [25,62]. The altered parameters in fibromyalgia include several systems, detailed below.

Central nervous system (CNS) level. The neurotransmitters serotonin, norepinephrine, and dopamine, implicated in the depression process, are altered in fibromyalgia, leading to decreased production. This explains the dual nature of fibromyalgia, often accompanied by depression, and the efficacy of antidepressant medications in treating fibromyalgia [11,63,64].

Peripheral sensitization level. Elevated levels of neurotransmitters such as glutamate and substance P are found in fibromyalgia [39,59]. Glutamate is an excitatory pronociceptive neurotransmitter associated with pain receptors. It was observed that in a subgroup of FM patients, treatment with N-methyl-D-aspartate (NMDA) receptor antagonists improved symptoms and pain perception [22]. However, the use of these drugs is not always well tolerated and may not always have clinical use. Substance P is a substance that promotes pain by binding to its NK1 receptor, found postsynaptically in the dorsal horn of the spinal cord, as well as in immune cells, smooth muscle cells, blood vessels, and other peripheral cells. In vitro studies conducted on these tissues similarly showed that blocking the binding to its receptor reduced this hyperalgesia [39].

Genetic level. Among the genes that may influence the onset of the condition is the TAAR1 gene, which mediates the availability of dopamine, a neurotransmitter crucial in the body’s motor function [65,66,67]. Its reduction may lead to the characteristic pain sensitivity increase of fibromyalgia. The *RGS4* gene, responsible for modulating the inhibition of descending pain perception pathways, is primarily expressed in the dorsal horn of the spinal cord [65,67]. Another gene associated with pain disorders is *CNR1* [68], responsible for encoding the cannabinoid receptor (CB-1), a G protein-coupled receptor. This gene controls and regulates limbic and reward processes in the brain and is found in both the central and peripheral nervous systems [65,67,69]. Finally, the *GRIA4* gene, implicated in the central sensitization phenomenon, is involved in the increased excitatory transmission of nociceptive signals reaching the central nervous system [65,67].

Endocrine level. The hypothalamic–pituitary–adrenal (HPA) axis links emotional and psychological factors with neuroendocrine production. Dysfunction of the HPA axis leads to an increase in basal adrenocorticotropic hormone (ACTH) levels and secretion in response to stress [70,71]. Additionally, individuals with fibromyalgia show a decrease in growth hormone, thyroxine, growth factor, estrogen, and cortisol. A study observed the direct and linear relationship between salivary cortisol levels in fibromyalgia subjects and their pain symptoms, indicating the impact of the patient’s mood on the disease. These elevated cortisol levels are indicative of high stress levels [70,72].

Psychological level. Psychological disorders are significant in fibromyalgia development, with a close relationship between stress [15,39,70], anxiety [11,63,64], depression [56,58,59,61], and worse prognosis of the condition. Stress has been shown to act as a predictive factor and worsen the prognosis of fibromyalgia. Stress can modulate pain sensitivity perception by inducing allodynia and hyperalgesia through physiological cholesterol secretion alteration, indirectly leading to cytokine release and triggering the previously described inflammatory and immune processes. Changes were also observed in processes occurring in the spinal cord, such as a reduction in both basal and induced release of the inhibitory neurotransmitter GABA and a reduction in antinociception of the mu opioid agonist, improving basal and evoked glutamate release, suggesting both increased central excitability and reduced central inhibition [5,22,40].

Sleep disorders. Sleep disturbances have been included as part of the symptoms and clinical manifestations of fibromyalgia patients [6,7,11]. Recent studies suggest they may be more related to a potential factor that could induce the development of the condition. Biochemical analyses suggest that insufficient sleep and rest may promote pain onset through an increase in serum IL-6 concentration [73]. Disrupted sleep continuity is considered more impactful in these analyses than sleep loss itself. Studies have also been conducted on sleep cycle structure, supporting the idea that sleep disturbances cause fibromyalgia development [6,7,11]. Specifically, subjects with this condition were observed to have a predominance of the sleep spindle phase. However, it should be noted that healthy individuals experiencing sleep disorders inherently trigger musculoskeletal pain processes. Moreover, an extensive study has been conducted on the anatomical substrate of sleep spindles [74]. These are sequences of electroencephalographic waves recurring every 3 to 10 s, with a frequency ranging from 12 to 16 Hz and a duration of 0.5 to 1.5 s. They are characteristic of non-REM sleep [75]. Sleep spindles are produced by the rhythmic discharge of thalamic relay neurons that induce the onset and maintenance of non-restorative sleep, causing interruptions in sleep continuity [76]. This leads to stress, anxiety, and depression disorders, thus correlating with fibromyalgia onset.

## 5. Diagnosis

### 5.1. Bases and Diagnostic Advances

The diagnosis and assessment of fibromyalgia remain controversial [12,77,78]. Over time, various alternative diagnostic methods for this condition have emerged, forming the basis of the most recent advances. In general, most researchers agree on the need to assess multiple domains of fibromyalgia, including pain, sleep, fatigue, concentration problems, anxiety, overall functional status, sensitivity, and stiffness. The American College of Rheumatology (ACR) initially suggested the 1990 diagnostic criteria based on the assessment of generalized pain defined as pain present on both sides of the body at upper and lower waist levels, in addition to axial skeletal pain, and on the assessment of pain sensitivity to palpation in more than 11 of the 18 established tender points, excluding the rest of the symptoms also present in this condition [77]. Tender points are areas of high sensitivity to mechanical stimuli, meaning they have a low threshold for mechanical pain. This ACR 1990 criterion is not practical clinically as the examination of tender points is not entirely objective for both the examiner and the examined. The 2010–2011 diagnostic criteria interpret fibromyalgia as a syndrome of multiple symptoms, thus eliminating the examination of tender points. Although these criteria evaluate fibromyalgia more generally, they place little emphasis on chronic pain, the main symptom of the condition, which greatly affects the patient. They were based on the use of the widespread pain index (WPI) combined with the severity scale of symptoms (SSS). The widespread pain index is based on the sum of body regions perceived as painful by the patient over the past week, ranging from 0 to 19 regions. The severity scale of symptoms (SSS) is based on the sum of fatigue severity, unrefreshed waking, and symptoms affecting cognitive and somatic levels on a scale from 0 to 1.

**Figure 3:**
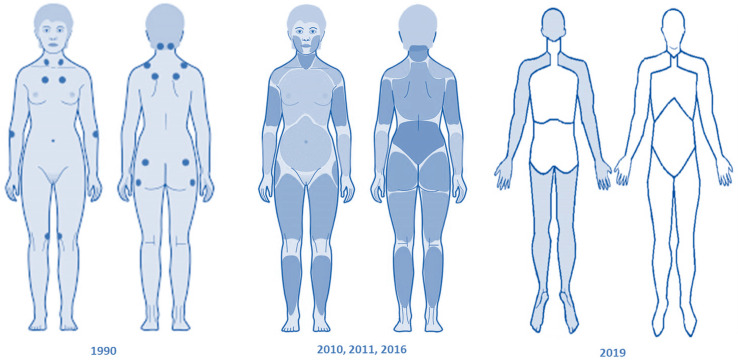
Evolution of classification criteria for diagnosing fibromyalgia across different years. The diagnosis and assessment of fibromyalgia have evolved over time. The American College of Rheumatology initially proposed criteria focusing on generalized pain and pain sensitivity. The 2010–2011 criteria interpreted fibromyalgia as a syndrome of multiple symptoms, emphasizing widespread pain and symptom severity. In 2016, a review incorporated four criteria: widespread pain, generalized pain, symptoms duration, and pain and symptom severity scores.

In 2016, a new review of the 2010–2011 fibromyalgia criteria was conducted based on widespread pain and clinical data, incorporating the following four criteria: presence of widespread pain in at least four of five regions, symptoms present at a similar level and intensity for a minimum of three months, a widespread pain index (WPI) score greater than or equal to 7 and a severity scale of symptoms (SSS) score greater than or equal to 5, or a WPI score of 4–6 and an SSS score greater than or equal to 9. Lastly, it was emphasized that the diagnosis of fibromyalgia should not be excluded by the presence of other clinically significant diseases. Subsequently, the basic diagnostic criteria of AAPT (ACTTION-American Pain Society Pain Taxonomy) were included, which used the multisite pain scale (MSP) to detect pain based on a count from 0 to 9 of the number of body regions that have been painful for at least three months. Additionally, moderate and severe sleep problems were evaluated, as well as the presence of moderate or intense fatigue [12,77]. Salaffi et al. conducted a study comparing the agreement of ACR 2011, ACR 2016, and AAPT criteria in fibromyalgia diagnosis [79]. The study showed that ACR 2011 criteria provided the best performance, while AAPT criteria offered the worst. With this study, an additional set of criteria based on the Modified Fibromyalgia Assessment Status (FAS 2019 modified) were developed, which provided a diagnostic accuracy comparable to the evaluated diagnostic criteria [79,80,81,82] (Figure 3).

More recently, the position statement of the Italian Society of Neurology’s Neuropathic Pain Study Group (NPSG) [20] has proposed practical guidelines with an innovative approach that integrates the traditional clinical assessment with new techniques investigating the sensory system in fibromyalgia, with the aim of refining the diagnostic workup as well as offering further insight into pathophysiology [83]. An emerging point is that many fibromyalgia patients display decreased intraepidermal nerve fiber density on skin biopsy and other functional and/or structural signatures of small fiber involvement.

### 5.2. Diagnostic Biomarkers

The main challenge in diagnosing this condition is the scarcity of biomarkers. Currently, there are no specific biomarkers, so research focuses on studying new indicators to obtain an objective diagnosis of affected individuals through the identification of environmental, genetic, and epigenetic factors underlying the pathophysiology of fibromyalgia.

#### 5.2.1. Genetic Biomarkers

Several studies conducted in family groups and the prevalence obtained therein support the theory that genetic factors, along with environmental factors such as diseases, trauma, or emotional stress, could predispose individuals to fibromyalgia [69]. Genetic polymorphisms implicated in mood disorders are considered risk factors [18,66,69]. These genes include candidates such as the serotonin transporter gene (5-HTT), the serotonin receptor 2A (HT2A), catechol-O-methyltransferase (COMT), and the dopamine receptor. Other genes associated with fibromyalgia that regulate nociceptive and analgesic neuronal pathways are the TAAR1 receptor gene, the regulator of G protein signaling 4 gene (RGS4), the cannabinoid receptor 1 gene (CNR1), and the ionotropic glutamate receptor AMPA 4 gene (GRIA4) [65,67].

#### 5.2.2. Serological Biomarkers

There is considerable interest in diagnosing fibromyalgia through blood analysis, leading to research efforts to find unique serological markers related to this condition. However, findings from these studies, like those from genetic tests, are frequently contradictory, and clinical evidence has not been confirmed thus far. Recently, analyses performed on blood samples from fibromyalgia patients revealed the role of the mu opioid receptor in B lymphocytes as a specific biomarker for fibromyalgia diagnosis [66]. These studies found a lower percentage of mu-positive B lymphocytes in subjects with the condition compared to healthy individuals. Therefore, this receptor could be used as a biomarker to obtain an objective diagnosis of chronic pain, such as that experienced by fibromyalgia patients. More recently, the evaluation of neutrophil/lymphocyte, lymphocyte/monocyte, and monocyte/high-density lipoprotein ratios in patients with fibromyalgia has allowed the determination of their relationship with disease activity, pain, and depression levels [84].

In our laboratory, we assayed the utility of several biomarkers in fibromyalgia patients [41,42,43,85]. Here, fibromyalgia patients showed higher serum levels of aspartic acid, glutamic acid, aminoadipic acid, asparagine, histidine, 3-methyl-histidine, 5-methyl-histidine, glycine, threonine, taurine, tyrosine, valine, methionine, isoleucine, phenylalanine, leucine, ornithine, lysine, branched chain AAs (BCAAs), large neutral AAs, essential AAs (EAAs), non-essential AAs (NEAAs), and basic AAs as well as a higher EAA/NEAA ratio, phenylalanine/tyrosine ratio, and global arginine bioavailability ratio than the controls. Serum alanine levels were lower in patients than in controls. According to the ROC analysis, most of these AAs may be good markers for differentiating individuals with fibromyalgia from healthy subjects. The results of the logistic regression showed that the combination of glutamic acid, histidine, and alanine had the greatest predictive ability to diagnose fibromyalgia [41]. These results demonstrate the imbalance in serum of most AAs in patients with fibromyalgia, which suggests a metabolic disturbance. Determining the serum levels of these AAs may aid in the diagnosis of fibromyalgia, in combination with clinical data of the patient [41].

We also measured plasma catecholamines (epinephrine, norepinephrine, and dopamine) as well as indolamines and intermediary metabolites (serotonin or 5-hydroxytryptamine [5-HT], 5-hydroxyindolacetic acid [5-HIAA], 5-hydroxytryptophan [5-HTP], and N-acetyl-5-hydroxytryptamine [Nac-5-HT]) in women with fibromyalgia and age-matched healthy women. Higher levels of norepinephrine and lower levels of dopamine, 5-HT, 5-HIAA, and 5-HTP were found in women with fibromyalgia in comparison with controls. Epinephrine and Nac-5-HT levels did not differ significantly between groups. Higher norepinephrine levels were associated with worse physical health status in fibromyalgia patients. Additionally, plasma norepinephrine levels > 694.69 pg/mL might be an accurate predictor of fibromyalgia. These findings show evidence of the dysregulation of the catecholamine and indolamine pathway in patients with fibromyalgia, which may contribute to the physiopathology of this syndrome. In addition, determining the plasma norepinephrine levels could help in diagnosis [42].

In other studies of ours, we found that oxytocinase activity was higher in patients with fibromyalgia than in controls. A subgroup of patients with fibromyalgia showed low levels of enkephalin-degrading aminopeptidase (EDA) activity when compared with the healthy controls and with the other fibromyalgia patients. There were no significant differences in the activity levels of aminopeptidase A, aminopeptidase B, aspartyl aminopeptidase, insulin-regulated aminopeptidase, pyroglutamyl aminopeptidase, or aminopeptidase N between fibromyalgia patients and controls. According to the ROC analysis, oxytocinase activity may be a good marker for differentiating individuals with fibromyalgia from healthy subjects. We concluded that that serum oxytocinase activity is increased in patients with fibromyalgia, which could alter the metabolism of peptides with analgesic effects such as oxytocin and enkephalins. Determining serum oxytocinase activity may also aid in fibromyalgia diagnosis. Additionally, we identified a subpopulation of fibromyalgia patients with abnormally low serum EDA activity [43]. Furthermore, we performed an observational case study in a population of women diagnosed with fibromyalgia. Serum nitric oxide levels were analyzed by an ozone chemiluminescence-based assay. Both serum oxytocinase and EDA activities were fluorometrically determined. Pain threshold and pain magnitude were evaluated using the PainMatcher instrument (Cefar Medical AB, Lund, Sweden). The pressure pain thresholds were measured using a digital pressure algometer. We used a visual analog scale, the Central Sensitization Inventory, the Revised Fibromyalgia Impact Questionnaire, and the Beck Anxiety Inventory to assess the global level of pain, the symptoms associated with the central sensitization syndrome, the severity of fibromyalgia, and the anxiety level, respectively. Multiple linear regression analysis adjusted by age, body mass index, and menopause status revealed significant associations between nitric oxide levels and dominant occiput pressure pain thresholds, nondominant occiput pressure pain thresholds, and fibromyalgia effects. Significant associations of oxytocinase activity with the visual analog scale and dominant knee pressure pain thresholds were also found. Moreover, the results showed a significant association between high EDA activity levels and dominant second-rib pressure pain thresholds. Our data have shown significant relationships of serum nitric oxide levels and oxytocinase and EDA activities with some body pressure pain thresholds, daily activity level, and the global intensity of pain in women with fibromyalgia. These results suggest that pain, which is the main symptom of this syndrome, may be related to alterations in nitric oxide levels and in oxytocinase and EDA activities in patients with fibromyalgia [85].

#### 5.2.3. Role of Vibrational Spectroscopy in Fibromyalgia

Recent studies have explored the utility of vibrational spectroscopy techniques, specifically Fourier-transform infrared spectroscopy (FT-IR) and Raman spectroscopy, in addressing the diagnostic gaps of fibromyalgia. Thus, a study aimed to develop a rapid diagnostic method using portable FT-IR spectroscopy to differentiate fibromyalgia from systemic lupus erythematosus (SLE), osteoarthritis (OA), and rheumatoid arthritis (RA). Bloodspot samples from fibromyalgia patients and individuals with related rheumatologic disorders were analyzed. The spectral data collected were subjected to pattern recognition analysis, specifically Orthogonal Projections to Latent Structures Discriminant Analysis (OPLS-DA), achieving high classification accuracy. Key spectral features such as aromatic amino acids and peptide backbones were identified as discriminatory biomarkers for fibromyalgia [86]. FT-IR and FT-Raman microspectroscopy coupled with metabolomics analysis also allowed to differentiate fibromyalgia from RA, OA, and SLE in a study utilizing advanced metabolomic techniques, including ultra-high performance liquid chromatography coupled to photodiode array detection and tandem mass spectrometry (uHPLC-PDA-MS/MS), to characterize metabolic differences. Unique IR and Raman spectral signatures associated with fibromyalgia were identified through multivariate analysis, demonstrating significant interclass distances and robust correlations with fibromyalgia pain severity as measured by the fibromyalgia impact questionnaire, revised version (FIQR) [87,88].

## 6. Treatment

Given that fibromyalgia is a multifactorial disease, its treatment, as expected, is also multifaceted. In other words, it encompasses multiple disciplines, yet unfortunately, none achieve the ultimate goal that every healthcare professional aims for with a disease: a cure.

### 6.1. Pharmacological Treatment

The pharmacological treatment of fibromyalgia not only addresses nociceptive symptoms but also treats associated symptoms such as depression, anxiety, and sleep disturbances. It is worth noting that monotherapy drug treatments for fibromyalgia often fail to achieve the desired goals for patients. Instead, combinations of various drugs, as demonstrated in several studies, tend to better alleviate symptoms of this condition. Several therapeutic groups are used to treat the signs and symptoms of fibromyalgia, as detailed below.

Cannabinoids. These drugs bind to CB1 and CB2 receptors, producing analgesic effects in fibromyalgia. Currently, their use is not authorized in most countries, and there are no commercial presentations available in Spain. Due to the serious adverse drug reactions they can cause, their use in treatment is not recommended, as the risks outweigh the benefits obtained in pain management [40,68].

Opioids. These drugs, derived from opium obtained from the *Papaver somniferus* plant, are currently used to treat pain in fibromyalgia [40]. Tramadol is the most commonly used opioid in fibromyalgia treatment [89]. Patients who took tramadol reported a decrease in their pain levels on the scale. However, due to adverse reactions such as dizziness, drowsiness, anxiety, and constipation, it is sometimes reserved for refractory cases resistant to other treatments. As a last resort in treatment, more potent opioids than morphine, such as fentanyl, would be considered for extremely acute crises, as clinical studies have shown that they can inhibit the onset of severe secondary pain [90].

Gabapentinoids. Pregabalin is the most commonly used drug in this therapeutic group to treat fibromyalgia [5]. It is one of the most widely used drugs in the treatment of this condition, and its clinical utility for reducing pain has been demonstrated in numerous studies. Its mechanism of action is based on reducing the perception of sensory or neuropathic pain [91,92].

Antidepressants. Antidepressants are a therapeutic group of drugs that are useful in treating fibromyalgia both in terms of pain and emotional symptoms [89,93].

Serotonin and norepinephrine reuptake inhibitors. Mirtazapine, which increases the release of serotonin and noradrenaline, thus exerting antidepressant effects, appears to be one of the drugs that most helps fibromyalgia patients improve sleep, pain, and quality of life [93]. Duloxetine is also used to treat fibromyalgia in many countries and can produce greater pain relief in fibromyalgia patients than a placebo [94,95].

Tricyclic antidepressants (TCAs). TCAs represent a class of medications that have been utilized in the pharmacological management of fibromyalgia due to their analgesic and central nervous system modulating properties. The most representative and prescribed drug in this group is amitriptyline. Despite not having numerous studies to corroborate its efficacy, it is one of the drugs prescribed for fibromyalgia, and patients report improvement in pain and sleep quality [96,97]. Amitriptyline enhances central pain modulation through inhibition of norepinephrine and serotonin reuptake. Its sedative effects also contribute to improving sleep quality, which is frequently disrupted in fibromyalgia patients [98]. Nortriptyline, another TCA, exhibits a similar pharmacological profile to amitriptyline but with a potentially better tolerability profile due to its lower anticholinergic effects [99]. Other TCAs such as desipramine and imipramine have also been studied in fibromyalgia, although with varying degrees of efficacy and side effect profiles [98]. While TCAs offer benefits in alleviating pain and improving sleep in fibromyalgia, their use is limited by adverse effects such as dry mouth, constipation, and potential cardiac complications in susceptible individuals. Despite these limitations, TCAs remain an important pharmacological treatment for fibromyalgia, particularly in cases where other first-line treatments such as serotonin–norepinephrine reuptake inhibitors and gabapentinoids are either ineffective or not tolerated [99].

The aforementioned drugs can improve the quality of life of fibromyalgia patients but do not cure their symptoms.

### 6.2. Non-Pharmacological Treatments: Physical Therapy Treatment

#### 6.2.1. Exercise Therapy

Exercise therapy is considered a significant component in the treatment of fibromyalgia, as it has been shown to improve pain intensity, sleep disturbances, fatigue, and depression [92,100,101,102,103,104,105]. Therefore, engaging in physical exercise is linked to enhancing the quality of life for individuals with fibromyalgia, improving their ability to perform daily activities [106].

There are several physiological mechanisms that could explain the beneficial effect of physical exercise on fibromyalgia patients. These mechanisms are associated with the main issue of the pathology, the presence of chronic pain. Physical activity restores appropriate signaling of the hypothalamic–pituitary–adrenal (HPA) axis and structures at a higher level [107,108]. It has been demonstrated that exercise restores negative input in the hippocampus and restores the proper output of the HPA axis, as physical exercise increases dendritic complexity and the expression of brain-derived neurotrophic factor (BDNF) in the hippocampus, which is primarily involved in brain processes related to learning, memory, and cognition [65,109]. Additionally, physical exercise leads to the release of corticotropin-releasing factor (CRF), stabilizing chronic pain disorders and reducing the input of peripheral nociceptors [65,110]. It also influences the descending pain pathways through the release of endogenous opioids, both in excitatory signals with the release of the neurotransmitter glutamate and in inhibitory signals with the release of GABA [5,22,40,91,92,111]. 

A large body of literature shows the effectiveness of physical exercise on fibromyalgia symptomatology based on the Fibromyalgia Impact Questionnaire (FIQ) [112,113,114,115,116]. The FIQ is an assessment tool used to measure the progress, status, and outcomes of patients with fibromyalgia. It consists of 10 items. The first item is associated with questions related to physical functioning; items two and three are associated with fibromyalgia symptoms, indicating the proportion of time the patient has felt well and to what extent they have been limited by the symptoms produced by the condition; and finally, items four to ten contain linear scales based on increments of 10 in which the subject rates the difficulty they have experienced in working, pain, sleep disturbances, and fatigue as well as anxiety and depression.

Physical exercise presents different modalities depending on the physical objective pursued. Several types of physical exercise have been studied for the treatment of fibromyalgia, which are the most effective in reducing symptoms and improving the quality of life of patients.

Stretching exercises have shown improvement in physical function and pain, thus significantly impacting the patient’s quality of life, while resistance training influences the reduction of depression symptoms [116].

Aerobic exercise, in studies evaluated with the FIQ scale, has resulted in weak evidence related to pain intensity, increased motor function, and reduced depression symptoms; however, moderate evidence was obtained regarding the improvement of the patient’s quality of life [113,117].

Flexibility exercises are recommended to relieve muscle tension, increase muscle length, and thus generate greater joint range of motion. Studies have concluded that this type of exercise is effective in reducing pain, fatigue, and sleep disturbances, and thus improving the patient’s quality of life [118]. The results obtained in the FIQ scale may be altered by a lack of detailed protocols applied for flexibility exercises.

Another recommended exercise modality is muscle strengthening. Patients with fibromyalgia often have reduced strength, which decreases functionality. Studies on muscle strength training obtained weak evidence regarding the improvement of physical function, pain, muscle strength, and sensitivity on the FIQ scale [100,114]. These results were affected by the variability between treatment protocols in the studies.

Therapeutic Pilates practice has also been studied. It combines strength training exercises with flexibility exercises, in addition to incorporating aerobic and stretching exercises [119,120,121,122]. Pilates aims to improve overall body flexibility, posture, and core strength. Its effectiveness as a treatment was determined by significant results in improving aspects such as vitality, pain modulation, and functional capacity on the FIQ scale. Pilates practice is recommended for healthy individuals but can also lead to pain reduction and, therefore, improved quality of life in fibromyalgia patients. Another example of combined exercise was obtained in a study in which strength and flexibility exercises were included in aerobic exercise [123]. Improvements were observed in the range of motion of the shoulder and hip joints, as well as an increase in grip strength. Grip strength is considered an important factor in the patient’s health and their predisposition to hospital stays [124].

Each of the explained exercise modalities provides benefits in reducing fibromyalgia symptoms. Therefore, a highly effective treatment for patients’ clinical manifestations through physical exercise is based on a functional and individualized training program for each patient. A long-term training program was carried out with a frequency of three times per week in sessions lasting 30 to 60 min. After the follow-up period, it was observed that the active group experienced an improvement in leg strength, grip strength, balance, and cardiorespiratory capacity compared to the control group. Additionally, very significant improvements in the quality of life of the subjects were noted.

#### 6.2.2. Hydrotherapy

Hydrotherapy or aquatic therapy has proven to be another effective discipline in the treatment of fibromyalgia as it involves performing physical exercise in water, improving the symptoms associated with the condition. Hydrotherapy takes advantage of the hydrodynamic properties provided by the aquatic environment, such as its density, viscosity, buoyancy, and hydrostatic pressure. These properties provide resistance to movement, thus working on joint strengthening, increasing muscle relaxation, and consequently reducing the impact on joints during movement. Additionally, exercises in the aquatic environment improve venous return [104,125,126,127,128,129]. Pain scores on the FIQ scale showed significant improvements in sleep quality and pain perception with aquatic aerobic exercise.

#### 6.2.3. Electrotherapy

Electrotherapy is an electrical therapeutic modality for muscle or brain region stimulation used to obtain analgesia in fibromyalgia patients [130,131,132,133]. Subjects with this condition not only experience pain, fatigue, and sleep disturbances, among other symptoms, but also suffer from cognitive impairment. Cognitive impairments contribute to a sense of confusion and slowness in patients, greatly impacting their ability to organize and execute daily activities. Several techniques have been established as effective for the treatment of this condition, although they continue to be studied. One studied invasive electrotherapy technique is occipital nerve stimulation through direct current with subcutaneous electrodes, which is related to the inhibition of the pain descending pathway [134,135].

Non-invasive brain stimulation has resulted in effective modulation of certain brain areas. This stimulation is carried out using electric or magnetic currents applied to the scalp as transcranial stimulation. Transcranial direct current stimulation is applied at the primary motor cortex, the main area of the brain responsible for producing nerve impulses for voluntary movements. This technique can lead to changes in excitability and neuronal neuroplasticity. Transcranial magnetic stimulation over the dorsolateral prefrontal cortex, responsible for cognitive control, has been shown to have effects on the excitatory modulation of cortical and deep brain areas through current pulses [136,137,138,139,140,141,142,143].

Considering that pharmacological and non-pharmacological treatments are often ineffective or transient in their effect on fibromyalgia, therapeutic electrical stimulation appears to have a potential role in its management.

#### 6.2.4. Manual Therapy

Manual therapy is defined as a physiotherapy technique administered manually, including low- or high-velocity joint techniques, stretching, and soft tissue manipulation or myofascial release techniques [144,145,146,147,148]. There has been an increasing focus on manual therapy as a treatment for fibromyalgia as it acts on nociceptive pathways at an ascending level considered to be related to central sensitization, resulting in pain improvement [144,145,146,148]. A recent study focused on evaluating the effectiveness of a manual therapy technique utilizing moderate digital pressure in women diagnosed with FM. The results revealed a significant decrease in pain perception as well as an improvement in muscle fatigue and tension-anxiety state following the manual therapy sessions compared to a placebo group. Additionally, correlations were identified between fatigue and sleep disturbances as well as between pain and anger-hostility subscales in the mood profile, highlighting the intricate interplay of FM symptoms and the importance of comprehensive management [145,146,148]. Another therapeutic approach under investigation is manual lymphatic drainage and myofascial therapy. A systematic review of randomized clinical trials compared the outcomes of both therapies in FM patients. While improvements in quality of life were observed across all included studies, the available evidence was rated as ranging from very weak to moderate due to result variability. This underscores the necessity for further research to better understand the efficacy of these interventions and establish clear recommendations for their clinical application [144,147]. Conversely, a broader systematic review examined the efficacy of manual therapy in FM patients through a comprehensive analysis of published randomized clinical trials. The findings of this review were less conclusive, suggesting that current evidence is insufficient to support the widespread use of manual therapy in this population. However, it was noted that general osteopathic treatment was the sole intervention demonstrating clinically relevant improvements in pain relief compared to the control group [62,116,144]. Collectively, these findings underscore the importance of ongoing investigation into diverse therapeutic approaches to address various aspects of FM. While some promising interventions such as manual therapy and lymphatic drainage have been identified, a deeper understanding of their efficacy and underlying mechanisms is warranted. Furthermore, it is essential to consider the heterogeneity of the FM patient population and develop individualized therapeutic approaches to achieve optimal clinical outcomes and enhance the quality of life for those affected by this complex condition. However, there is still a gap in its efficacy compared to other practical approaches as manual therapy has nonspecific effects, such as the placebo effect. Although positive results have been found regarding the symptomatology of the condition, they have not been entirely conclusive.

## 7. Conclusions

Fibromyalgia is a complex and multifaceted condition that presents significant challenges in both diagnosis and treatment. Throughout the literature, it is evident that there is ongoing debate and exploration regarding various aspects of fibromyalgia, including its etiology, diagnosis, and management. Despite the progress made in understanding the condition, many aspects remain contentious, necessitating a comprehensive approach to address the diverse needs of patients. Thus, the diagnosis of fibromyalgia continues to be a subject of controversy, with evolving criteria and assessment methods reflecting the complex nature of the condition. Historically, the emphasis was placed on tender point examination, as outlined in the 1990 American College of Rheumatology criteria. However, these criteria have been criticized for their subjectivity and lack of emphasis on other key symptoms. Subsequent revisions, such as the 2010–2011 diagnostic criteria, shifted towards a more holistic assessment of fibromyalgia as a syndrome of multiple symptoms, considering factors beyond tender points. Despite these advancements, challenges remain in achieving an objective and universally accepted diagnostic approach, highlighting the need for ongoing research and refinement. The quest for biomarkers in fibromyalgia represents a significant area of research aimed at enhancing diagnostic accuracy and elucidating underlying pathophysiological mechanisms. Genetic biomarkers, particularly polymorphisms associated with neurotransmitter regulation, have garnered attention for their potential role in predisposing individuals to fibromyalgia. Serological biomarkers have also been explored, albeit with conflicting findings and limited clinical utility thus far. The identification of reliable biomarkers remains elusive, underscoring the complex interplay of genetic, environmental, and epigenetic factors in fibromyalgia pathogenesis. Pharmacological management of fibromyalgia encompasses a diverse array of medications targeting various symptoms, including pain, depression, anxiety, and sleep disturbances. While no single medication has demonstrated unequivocal efficacy in achieving symptom relief, combination therapy and individualized approaches have shown promise in improving patient outcomes. From cannabinoids to opioids, gabapentinoids, and antidepressants, each medication offers unique benefits and considerations, highlighting the importance of tailored treatment regimens based on patient-specific factors and preferences. Non-pharmacological interventions play a pivotal role in the comprehensive management of fibromyalgia, offering alternatives or complements to pharmacotherapy. Physical modalities such as exercise therapy, hydrotherapy, and electrotherapy have demonstrated beneficial effects in reducing pain, improving functional status, and enhancing quality of life. Additionally, manual therapy techniques have shown promise in addressing nociceptive pathways and central sensitization, although further research is needed to elucidate their efficacy fully. The integration of non-pharmacological interventions into treatment plans underscores the multifaceted nature of fibromyalgia management, emphasizing the importance of personalized and multimodal approaches.
